# {9-Hexyl-2-[2-phenyl-6-(pyridin-2-yl)pyridin-4-yl]-9*H*-carbazole}di­iodido­zinc

**DOI:** 10.1107/S1600536813025464

**Published:** 2013-09-21

**Authors:** Hui Wang, Xue-Song Zhao, Jun-Shan Luo, Yu-Peng Tian

**Affiliations:** a Department of Chemistry, Anhui University, Hefei 230039, People’s Republic of China, and Key Laboratory of Functional Inorganic Materials, Chemistry, Hefei 230039, People’s Republic of China

## Abstract

In the title compound, [ZnI_2_(C_34_H_31_N_3_)], the Zn^II^ atom is four-coordinated by two I atoms and the pyridine N atoms from the bidentate 6′-phenyl-2,2′-bi­pyridine ligand in a distorted tetra­hedral geometry.

## Related literature

For the synthesis of the title compound and related structures, see: Alizadeh *et al.* (2009 ▶); Gao *et al.* (2009 ▶); Prokhorov *et al.* (2011 ▶).
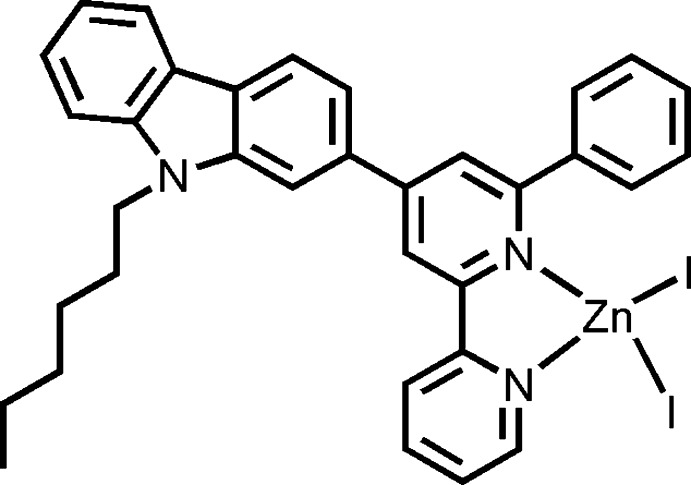


## Experimental

### 

#### Crystal data

[ZnI_2_(C_34_H_31_N_3_)]
*M* *_r_* = 800.79
Monoclinic, 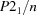

*a* = 15.3870 (14) Å
*b* = 9.8771 (9) Å
*c* = 21.3246 (19) Å
β = 99.306 (1)°
*V* = 3198.2 (5) Å^3^
*Z* = 4
Mo *K*α radiation
μ = 2.73 mm^−1^
*T* = 296 K
0.22 × 0.22 × 0.21 mm

#### Data collection

Bruker SMART CCD area-detector diffractometer
Absorption correction: multi-scan (*SADABS*; Sheldrick, 2002 ▶) *T*_min_ = 0.586, *T*_max_ = 0.598
23438 measured reflections
6226 independent reflections
5325 reflections with *I* > 2σ(*I*)
*R*_int_ = 0.022

#### Refinement

*R*[*F* ^2^ > 2σ(*F* ^2^)] = 0.039
*wR*(*F* ^2^) = 0.122
*S* = 1.04
6226 reflections
362 parameters
H-atom parameters constrained
Δρ_max_ = 1.10 e Å^−3^
Δρ_min_ = −0.92 e Å^−3^

### 

Data collection: *SMART* (Bruker, 2002 ▶); cell refinement: *SAINT* (Bruker, 2002 ▶); data reduction: *SAINT*; program(s) used to solve structure: *SHELXS97* (Sheldrick, 2008 ▶); program(s) used to refine structure: *SHELXL97* (Sheldrick, 2008 ▶); molecular graphics: *SHELXTL* (Sheldrick, 2008 ▶); software used to prepare material for publication: *SHELXTL*.

## Supplementary Material

Crystal structure: contains datablock(s) I, Global. DOI: 10.1107/S1600536813025464/hp2061sup1.cif

Structure factors: contains datablock(s) I. DOI: 10.1107/S1600536813025464/hp2061Isup2.hkl

Additional supplementary materials:  crystallographic information; 3D view; checkCIF report

## Figures and Tables

**Fig. 1.**
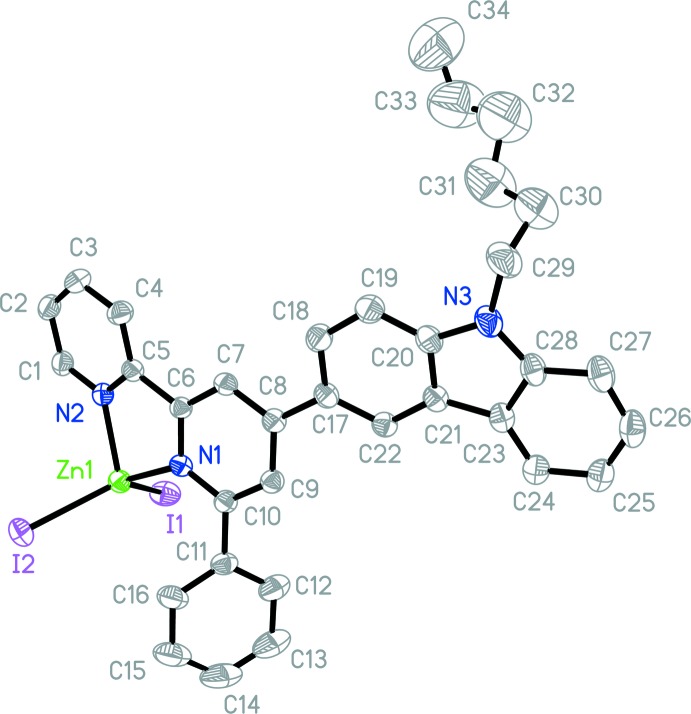
: The molecular structure of (I), with the atom-numbering scheme. Displacement ellipsoids are drawn at the 30% probability level. H atoms have been omitted.

## supplementary crystallographic information

### 1. Comment

In recent years, 6'-phenyl-2,2'-bipyridine based materials have attracted considerable interests because they have significant applications in optoelectronic functional materials (Prokhorov *et al.*, 2011). In addition, zinc complexes are particularly attractive and most studied for their biocompatibility (Gao *et al.*, 2009). Herewith, in this study, we report the crystal structure of the title compound (I).

In (I) (Fig.1), the Zn^II^ atom is four-coordinated by two I atoms and the N atoms from 6'-phenyl-2,2'-bipyridine rings in a distorted tetrahedral geometry and with the coordinated pyridine moities oriented in an almost coplanar fashion with a dihedral angle of 12.68 (1)°, which is larger than what is reported in the literature, with formula [ZnCl_2_(C_12_H_12_N_2_)] (II) (7.57°) (Alizadeh *et al.*, 2009), the reason is that the introduction of benzene increases steric hindrance. Zn—I bond distances are 2.5396 (6) and 2.5623 (6) Å, which are within normal range. Compared to (II),the distances of Zn—N are a little larger. I—Zn—I and N—Zn—N bond angles are 118.56 (2)° and 80.1 (1)°, which is smaller than that of (II), respectively.

### 2. Experimental

A solution of 9-hexyl-2-(2-phenyl-6-(pyridin-2-yl)pyridin-4-yl)-9*H*-carbazole (0.48 g, 1 mmol) in methanol (20 ml) was mixed with a zinc iodide (0.32 g, 1 mmol) in methanol (5 ml) and the reaction mixture was reflux for 4 h. The reaction mixture was cooled to room temperature and filtered into a large test tube. The light yellow crystals were obtained at room temperature after a week. Yield: 85%. ^1^H NMR (400 MHz, DMSO-d_6_) 8.89(s, 1H), 8.78–8.80(t, 2H), 8.66–8.68(d, 1H), 8.38–8.44 (m, 4H), 8.03–8.10 (q, 2H), 7.76–7.78 (d, 1H), 7.49–7.66 (q, 6H), 7.25–7.29 (t, 1H), 4.39–4.42 (t, 2H), 1.76–1.82 (q, 2H), 1.19–1.32 (q, 6H), 0.79–0.82 (t, 3H).

### 3. Refinement

All hydrogen atoms were placed in geometrically idealized positions and constrained to ride on their parent atoms, with C—H = 0.93–0.97 Å and *U*_iso_(H) = 1.2–1.5 *U*_eq_.

### Crystal data

**Table:**

[ZnI_2_(C_34_H_31_N_3_)]	*F*(000) = 1568
*M**_r_* = 800.79	*D*_x_ = 1.663 Mg m^−^^3^
Monoclinic, *P*2_1_/*n*	Mo *K*α radiation, λ = 0.71073 Å
Hall symbol: -P 2yn	Cell parameters from 9968 reflections
*a* = 15.3870 (14) Å	θ = 2.3–26.0°
*b* = 9.8771 (9) Å	µ = 2.73 mm^−^^1^
*c* = 21.3246 (19) Å	*T* = 296 K
β = 99.306 (1)°	Block, yellow
*V* = 3198.2 (5) Å^3^	0.22 × 0.22 × 0.21 mm
*Z* = 4	

### Data collection

**Table:**

Bruker SMART CCD area-detector diffractometer	6226 independent reflections
Radiation source: fine-focus sealed tube	5325 reflections with *I* > 2σ(*I*)
Graphite monochromator	*R*_int_ = 0.022
phi and ω scans	θ_max_ = 26.0°, θ_min_ = 1.5°
Absorption correction: multi-scan (*SADABS*; Sheldrick, 2002)	*h* = −18→18
*T*_min_ = 0.586, *T*_max_ = 0.598	*k* = −12→11
23438 measured reflections	*l* = −23→26

### Refinement

**Table:**

Refinement on *F*^2^	Primary atom site location: structure-invariant direct methods
Least-squares matrix: full	Secondary atom site location: difference Fourier map
*R*[*F*^2^ > 2σ(*F*^2^)] = 0.039	Hydrogen site location: inferred from neighbouring sites
*wR*(*F*^2^) = 0.122	H-atom parameters constrained
*S* = 1.04	*w* = 1/[σ^2^(*F*_o_^2^) + (0.0673*P*)^2^ + 4.6302*P*] where *P* = (*F*_o_^2^ + 2*F*_c_^2^)/3
6226 reflections	(Δ/σ)_max_ = 0.020
362 parameters	Δρ_max_ = 1.10 e Å^−^^3^
0 restraints	Δρ_min_ = −0.92 e Å^−^^3^

### Special details

**Table:**

Geometry. All e.s.d.'s (except the e.s.d. in the dihedral angle between two l.s. planes) are estimated using the full covariance matrix. The cell e.s.d.'s are taken into account individually in the estimation of e.s.d.'s in distances, angles and torsion angles; correlations between e.s.d.'s in cell parameters are only used when they are defined by crystal symmetry. An approximate (isotropic) treatment of cell e.s.d.'s is used for estimating e.s.d.'s involving l.s. planes.
Refinement. Refinement of *F*^2^ against ALL reflections. The weighted *R*-factor *wR* and goodness of fit *S* are based on *F*^2^, conventional *R*-factors *R* are based on *F*, with *F* set to zero for negative *F*^2^. The threshold expression of *F*^2^ > σ(*F*^2^) is used only for calculating *R*-factors(gt) *etc*. and is not relevant to the choice of reflections for refinement. *R*-factors based on *F*^2^ are statistically about twice as large as those based on *F*, and *R*- factors based on ALL data will be even larger.

### Fractional atomic coordinates and isotropic or equivalent isotropic displacement parameters (Å^2^)

**Table:**

	*x*	*y*	*z*	*U*_iso_*/*U*_eq_	
C1	0.0246 (3)	0.1788 (4)	−0.0159 (2)	0.0519 (9)	
H1	0.0503	0.1253	−0.0437	0.062*	
C2	−0.0605 (3)	0.1523 (5)	−0.0083 (2)	0.0586 (11)	
H2	−0.0923	0.0833	−0.0312	0.070*	
C3	−0.0979 (3)	0.2296 (5)	0.0339 (2)	0.0625 (11)	
H3	−0.1553	0.2129	0.0403	0.075*	
C4	−0.0492 (3)	0.3323 (5)	0.0668 (2)	0.0576 (10)	
H4	−0.0731	0.3843	0.0961	0.069*	
C5	0.0354 (2)	0.3570 (4)	0.05571 (18)	0.0438 (8)	
C6	0.0905 (2)	0.4704 (4)	0.08513 (17)	0.0415 (8)	
C7	0.0553 (3)	0.5746 (4)	0.11583 (19)	0.0448 (8)	
H7	−0.0041	0.5726	0.1196	0.054*	
C8	0.1077 (3)	0.6834 (4)	0.14154 (18)	0.0462 (8)	
C9	0.1963 (3)	0.6783 (4)	0.13391 (19)	0.0467 (8)	
H9	0.2342	0.7473	0.1506	0.056*	
C10	0.2285 (3)	0.5719 (4)	0.10179 (18)	0.0451 (8)	
C11	0.3224 (3)	0.5669 (5)	0.0938 (2)	0.0541 (10)	
C12	0.3621 (4)	0.6800 (6)	0.0716 (3)	0.0770 (15)	
H12	0.3304	0.7598	0.0628	0.092*	
C13	0.4498 (4)	0.6728 (9)	0.0627 (4)	0.101 (2)	
H13	0.4766	0.7482	0.0481	0.121*	
C14	0.4964 (4)	0.5559 (10)	0.0754 (4)	0.106 (2)	
H14	0.5541	0.5510	0.0679	0.127*	
C15	0.4591 (4)	0.4466 (9)	0.0991 (4)	0.099 (2)	
H15	0.4922	0.3684	0.1089	0.118*	
C16	0.3711 (3)	0.4505 (6)	0.1089 (3)	0.0740 (14)	
H16	0.3460	0.3756	0.1254	0.089*	
C17	0.0706 (3)	0.7983 (4)	0.17284 (19)	0.0484 (9)	
C18	−0.0098 (3)	0.7825 (5)	0.1959 (2)	0.0605 (11)	
H18	−0.0373	0.6984	0.1927	0.073*	
C19	−0.0487 (3)	0.8889 (5)	0.2231 (3)	0.0673 (13)	
H19	−0.1017	0.8770	0.2380	0.081*	
C20	−0.0068 (3)	1.0139 (5)	0.2275 (2)	0.0570 (10)	
C21	0.0745 (3)	1.0319 (4)	0.20552 (18)	0.0493 (9)	
C22	0.1119 (3)	0.9250 (4)	0.17774 (19)	0.0480 (9)	
H22	0.1645	0.9371	0.1623	0.058*	
C23	0.1006 (3)	1.1708 (4)	0.21957 (19)	0.0524 (10)	
C24	0.1738 (3)	1.2478 (5)	0.2116 (2)	0.0574 (10)	
H24	0.2179	1.2109	0.1918	0.069*	
C25	0.1801 (4)	1.3793 (5)	0.2336 (2)	0.0652 (12)	
H25	0.2290	1.4312	0.2287	0.078*	
C26	0.1142 (4)	1.4352 (5)	0.2630 (2)	0.0734 (14)	
H26	0.1205	1.5239	0.2777	0.088*	
C27	0.0402 (4)	1.3642 (5)	0.2710 (2)	0.0717 (14)	
H27	−0.0040	1.4033	0.2900	0.086*	
C28	0.0344 (3)	1.2295 (5)	0.2492 (2)	0.0592 (11)	
C29	−0.1112 (5)	1.1590 (8)	0.2789 (4)	0.103 (2)	
H29A	−0.0959	1.2105	0.3178	0.123*	
H29B	−0.1342	1.0724	0.2902	0.123*	
C30	−0.1837 (7)	1.2325 (11)	0.2369 (5)	0.139 (3)	
H30A	−0.2280	1.2610	0.2617	0.167*	
H30B	−0.1600	1.3127	0.2195	0.167*	
C31	−0.2230 (9)	1.1466 (14)	0.1863 (7)	0.170 (4)	
H31A	−0.2207	1.0541	0.2017	0.204*	
H31B	−0.1877	1.1509	0.1526	0.204*	
C32	−0.3178 (10)	1.1793 (16)	0.1580 (8)	0.192 (5)	
H32A	−0.3558	1.1698	0.1899	0.231*	
H32B	−0.3225	1.2713	0.1419	0.231*	
C33	−0.3423 (11)	1.0840 (17)	0.1072 (8)	0.202 (6)	
H33A	−0.3278	1.1241	0.0687	0.243*	
H33B	−0.3053	1.0046	0.1164	0.243*	
C34	−0.4308 (11)	1.0396 (17)	0.0940 (8)	0.216 (6)	
H34A	−0.4374	0.9586	0.1177	0.324*	
H34B	−0.4465	1.0213	0.0494	0.324*	
H34C	−0.4687	1.1088	0.1061	0.324*	
I1	0.23267 (2)	0.40572 (4)	−0.090212 (16)	0.07186 (13)	
I2	0.27913 (2)	0.09387 (3)	0.057852 (19)	0.07051 (13)	
N1	0.1767 (2)	0.4681 (3)	0.07773 (14)	0.0428 (7)	
N2	0.0721 (2)	0.2792 (3)	0.01525 (15)	0.0450 (7)	
N3	−0.0309 (3)	1.1340 (4)	0.2530 (2)	0.0645 (10)	
Zn1	0.20504 (3)	0.31593 (5)	0.01641 (2)	0.04805 (14)	

### Atomic displacement parameters (Å^2^)

**Table:**

	*U*^11^	*U*^22^	*U*^33^	*U*^12^	*U*^13^	*U*^23^
C1	0.058 (2)	0.043 (2)	0.053 (2)	−0.0009 (17)	0.0039 (18)	−0.0010 (17)
C2	0.059 (3)	0.049 (2)	0.065 (3)	−0.0094 (19)	0.000 (2)	0.002 (2)
C3	0.051 (2)	0.062 (3)	0.075 (3)	−0.013 (2)	0.011 (2)	0.001 (2)
C4	0.050 (2)	0.059 (3)	0.066 (3)	−0.0048 (19)	0.015 (2)	−0.005 (2)
C5	0.0423 (19)	0.0442 (19)	0.045 (2)	0.0018 (15)	0.0064 (15)	0.0023 (16)
C6	0.0397 (18)	0.0434 (19)	0.0417 (19)	0.0004 (15)	0.0069 (14)	0.0030 (15)
C7	0.0416 (19)	0.047 (2)	0.046 (2)	0.0027 (15)	0.0089 (16)	0.0004 (16)
C8	0.048 (2)	0.047 (2)	0.043 (2)	0.0010 (16)	0.0088 (16)	−0.0027 (16)
C9	0.045 (2)	0.047 (2)	0.048 (2)	−0.0013 (16)	0.0063 (16)	−0.0034 (17)
C10	0.045 (2)	0.046 (2)	0.043 (2)	−0.0007 (16)	0.0039 (16)	−0.0042 (16)
C11	0.042 (2)	0.064 (3)	0.055 (2)	−0.0022 (18)	0.0069 (18)	−0.017 (2)
C12	0.061 (3)	0.088 (4)	0.085 (4)	−0.018 (3)	0.020 (3)	−0.013 (3)
C13	0.072 (4)	0.126 (6)	0.110 (5)	−0.035 (4)	0.027 (4)	−0.014 (4)
C14	0.055 (3)	0.147 (7)	0.116 (6)	−0.007 (4)	0.020 (3)	−0.033 (5)
C15	0.056 (3)	0.120 (5)	0.115 (5)	0.020 (4)	−0.001 (3)	−0.031 (4)
C16	0.049 (2)	0.084 (3)	0.086 (4)	0.012 (2)	0.001 (2)	−0.017 (3)
C17	0.051 (2)	0.049 (2)	0.046 (2)	0.0049 (17)	0.0113 (17)	−0.0038 (17)
C18	0.062 (3)	0.055 (2)	0.069 (3)	−0.005 (2)	0.024 (2)	−0.009 (2)
C19	0.062 (3)	0.067 (3)	0.080 (3)	−0.002 (2)	0.032 (2)	−0.012 (2)
C20	0.060 (2)	0.057 (3)	0.057 (2)	0.007 (2)	0.018 (2)	−0.0084 (19)
C21	0.054 (2)	0.051 (2)	0.042 (2)	0.0046 (18)	0.0069 (17)	−0.0038 (17)
C22	0.048 (2)	0.052 (2)	0.045 (2)	0.0027 (17)	0.0100 (16)	−0.0041 (17)
C23	0.066 (3)	0.051 (2)	0.0385 (19)	0.0046 (19)	0.0030 (18)	−0.0030 (17)
C24	0.069 (3)	0.055 (2)	0.046 (2)	0.000 (2)	0.0002 (19)	0.0023 (19)
C25	0.083 (3)	0.055 (3)	0.051 (2)	−0.006 (2)	−0.006 (2)	0.003 (2)
C26	0.103 (4)	0.053 (3)	0.059 (3)	0.004 (3)	−0.002 (3)	−0.005 (2)
C27	0.096 (4)	0.060 (3)	0.060 (3)	0.013 (3)	0.014 (3)	−0.010 (2)
C28	0.077 (3)	0.051 (2)	0.049 (2)	0.009 (2)	0.010 (2)	−0.0063 (19)
C29	0.104 (5)	0.102 (5)	0.112 (5)	0.018 (4)	0.047 (4)	−0.009 (4)
C30	0.138 (8)	0.139 (8)	0.144 (8)	0.020 (6)	0.031 (6)	−0.003 (7)
C31	0.170 (11)	0.174 (11)	0.163 (11)	0.014 (9)	0.020 (9)	−0.011 (9)
C32	0.185 (13)	0.199 (14)	0.188 (13)	0.008 (11)	0.017 (11)	−0.014 (12)
C33	0.195 (16)	0.211 (16)	0.197 (15)	0.000 (12)	0.019 (13)	−0.021 (12)
C34	0.212 (16)	0.219 (15)	0.215 (16)	−0.020 (14)	0.031 (13)	−0.012 (13)
I1	0.0582 (2)	0.0995 (3)	0.0634 (2)	0.00331 (16)	0.02620 (15)	0.00944 (16)
I2	0.0692 (2)	0.0546 (2)	0.0881 (3)	0.01550 (14)	0.01370 (17)	0.00181 (15)
N1	0.0402 (15)	0.0457 (17)	0.0419 (16)	0.0035 (13)	0.0048 (12)	−0.0004 (13)
N2	0.0466 (17)	0.0403 (16)	0.0478 (17)	0.0002 (13)	0.0066 (14)	−0.0011 (13)
N3	0.073 (2)	0.058 (2)	0.067 (2)	0.010 (2)	0.026 (2)	−0.0115 (19)
Zn1	0.0455 (3)	0.0479 (3)	0.0521 (3)	0.00290 (19)	0.01186 (19)	−0.0046 (2)

### Geometric parameters (Å, º)

**Table:**

C1—N2	1.342 (5)	C20—C21	1.416 (6)
C1—C2	1.370 (6)	C21—C22	1.382 (6)
C1—H1	0.9300	C21—C23	1.447 (6)
C2—C3	1.376 (7)	C22—H22	0.9300
C2—H2	0.9300	C23—C24	1.392 (7)
C3—C4	1.383 (6)	C23—C28	1.407 (6)
C3—H3	0.9300	C24—C25	1.379 (7)
C4—C5	1.382 (6)	C24—H24	0.9300
C4—H4	0.9300	C25—C26	1.390 (8)
C5—N2	1.346 (5)	C25—H25	0.9300
C5—C6	1.481 (5)	C26—C27	1.371 (8)
C6—N1	1.362 (5)	C26—H26	0.9300
C6—C7	1.376 (5)	C27—C28	1.408 (7)
C7—C8	1.402 (6)	C27—H27	0.9300
C7—H7	0.9300	C28—N3	1.390 (7)
C8—C9	1.400 (6)	C29—N3	1.454 (7)
C8—C17	1.477 (5)	C29—C30	1.499 (12)
C9—C10	1.389 (5)	C29—H29A	0.9700
C9—H9	0.9300	C29—H29B	0.9700
C10—N1	1.348 (5)	C30—C31	1.429 (14)
C10—C11	1.482 (6)	C30—H30A	0.9700
C11—C16	1.382 (7)	C30—H30B	0.9700
C11—C12	1.393 (7)	C31—C32	1.520 (16)
C12—C13	1.394 (8)	C31—H31A	0.9700
C12—H12	0.9300	C31—H31B	0.9700
C13—C14	1.363 (11)	C32—C33	1.439 (17)
C13—H13	0.9300	C32—H32A	0.9700
C14—C15	1.357 (11)	C32—H32B	0.9700
C14—H14	0.9300	C33—C34	1.416 (17)
C15—C16	1.404 (8)	C33—H33A	0.9700
C15—H15	0.9300	C33—H33B	0.9700
C16—H16	0.9300	C34—H34A	0.9600
C17—C22	1.399 (6)	C34—H34B	0.9600
C17—C18	1.411 (6)	C34—H34C	0.9600
C18—C19	1.382 (6)	I1—Zn1	2.5396 (6)
C18—H18	0.9300	I2—Zn1	2.5623 (6)
C19—C20	1.389 (7)	N1—Zn1	2.084 (3)
C19—H19	0.9300	N2—Zn1	2.074 (3)
C20—N3	1.380 (6)		
			
N2—C1—C2	122.5 (4)	C28—C23—C21	106.7 (4)
N2—C1—H1	118.7	C25—C24—C23	119.1 (5)
C2—C1—H1	118.7	C25—C24—H24	120.4
C1—C2—C3	118.7 (4)	C23—C24—H24	120.4
C1—C2—H2	120.7	C24—C25—C26	120.7 (5)
C3—C2—H2	120.7	C24—C25—H25	119.6
C2—C3—C4	119.3 (4)	C26—C25—H25	119.6
C2—C3—H3	120.4	C27—C26—C25	122.3 (5)
C4—C3—H3	120.4	C27—C26—H26	118.8
C5—C4—C3	119.5 (4)	C25—C26—H26	118.8
C5—C4—H4	120.3	C26—C27—C28	116.9 (5)
C3—C4—H4	120.3	C26—C27—H27	121.6
N2—C5—C4	120.8 (4)	C28—C27—H27	121.6
N2—C5—C6	115.7 (3)	N3—C28—C23	109.2 (4)
C4—C5—C6	123.5 (4)	N3—C28—C27	129.1 (5)
N1—C6—C7	122.2 (3)	C23—C28—C27	121.7 (5)
N1—C6—C5	116.1 (3)	N3—C29—C30	116.8 (7)
C7—C6—C5	121.6 (3)	N3—C29—H29A	108.1
C6—C7—C8	120.9 (4)	C30—C29—H29A	108.1
C6—C7—H7	119.5	N3—C29—H29B	108.1
C8—C7—H7	119.5	C30—C29—H29B	108.1
C9—C8—C7	115.9 (4)	H29A—C29—H29B	107.3
C9—C8—C17	122.4 (4)	C31—C30—C29	110.3 (10)
C7—C8—C17	121.7 (4)	C31—C30—H30A	109.6
C10—C9—C8	121.1 (4)	C29—C30—H30A	109.6
C10—C9—H9	119.4	C31—C30—H30B	109.6
C8—C9—H9	119.4	C29—C30—H30B	109.6
N1—C10—C9	121.8 (4)	H30A—C30—H30B	108.1
N1—C10—C11	117.2 (3)	C30—C31—C32	115.8 (12)
C9—C10—C11	121.0 (4)	C30—C31—H31A	108.3
C16—C11—C12	119.7 (5)	C32—C31—H31A	108.3
C16—C11—C10	120.2 (4)	C30—C31—H31B	108.3
C12—C11—C10	120.1 (4)	C32—C31—H31B	108.3
C11—C12—C13	119.5 (6)	H31A—C31—H31B	107.4
C11—C12—H12	120.2	C33—C32—C31	106.1 (14)
C13—C12—H12	120.2	C33—C32—H32A	110.5
C14—C13—C12	120.4 (7)	C31—C32—H32A	110.5
C14—C13—H13	119.8	C33—C32—H32B	110.5
C12—C13—H13	119.8	C31—C32—H32B	110.5
C15—C14—C13	120.5 (6)	H32A—C32—H32B	108.7
C15—C14—H14	119.8	C34—C33—C32	118.6 (17)
C13—C14—H14	119.8	C34—C33—H33A	107.7
C14—C15—C16	120.6 (7)	C32—C33—H33A	107.7
C14—C15—H15	119.7	C34—C33—H33B	107.7
C16—C15—H15	119.7	C32—C33—H33B	107.7
C11—C16—C15	119.2 (6)	H33A—C33—H33B	107.1
C11—C16—H16	120.4	C33—C34—H34A	109.5
C15—C16—H16	120.4	C33—C34—H34B	109.5
C22—C17—C18	119.1 (4)	H34A—C34—H34B	109.5
C22—C17—C8	121.1 (4)	C33—C34—H34C	109.5
C18—C17—C8	119.8 (4)	H34A—C34—H34C	109.5
C19—C18—C17	121.7 (4)	H34B—C34—H34C	109.5
C19—C18—H18	119.1	C10—N1—C6	118.1 (3)
C17—C18—H18	119.1	C10—N1—Zn1	128.1 (3)
C18—C19—C20	118.5 (4)	C6—N1—Zn1	113.0 (2)
C18—C19—H19	120.8	C1—N2—C5	119.2 (4)
C20—C19—H19	120.8	C1—N2—Zn1	126.4 (3)
N3—C20—C19	129.8 (4)	C5—N2—Zn1	114.0 (2)
N3—C20—C21	109.3 (4)	C20—N3—C28	108.6 (4)
C19—C20—C21	120.9 (4)	C20—N3—C29	126.5 (5)
C22—C21—C20	119.8 (4)	C28—N3—C29	125.0 (5)
C22—C21—C23	133.9 (4)	N2—Zn1—N1	80.09 (12)
C20—C21—C23	106.3 (4)	N2—Zn1—I1	110.98 (9)
C21—C22—C17	120.0 (4)	N1—Zn1—I1	113.09 (9)
C21—C22—H22	120.0	N2—Zn1—I2	103.73 (9)
C17—C22—H22	120.0	N1—Zn1—I2	121.83 (9)
C24—C23—C28	119.2 (4)	I1—Zn1—I2	118.56 (2)
C24—C23—C21	134.1 (4)		
			
N2—C1—C2—C3	−1.4 (7)	C21—C23—C24—C25	−176.8 (4)
C1—C2—C3—C4	0.7 (7)	C23—C24—C25—C26	−0.4 (7)
C2—C3—C4—C5	1.2 (7)	C24—C25—C26—C27	−0.6 (8)
C3—C4—C5—N2	−2.6 (7)	C25—C26—C27—C28	1.4 (8)
C3—C4—C5—C6	175.7 (4)	C24—C23—C28—N3	−178.9 (4)
N2—C5—C6—N1	−12.0 (5)	C21—C23—C28—N3	−0.9 (5)
C4—C5—C6—N1	169.7 (4)	C24—C23—C28—C27	0.2 (7)
N2—C5—C6—C7	165.4 (4)	C21—C23—C28—C27	178.2 (4)
C4—C5—C6—C7	−12.9 (6)	C26—C27—C28—N3	177.8 (5)
N1—C6—C7—C8	−0.5 (6)	C26—C27—C28—C23	−1.1 (7)
C5—C6—C7—C8	−177.8 (4)	N3—C29—C30—C31	71.8 (12)
C6—C7—C8—C9	−0.1 (6)	C29—C30—C31—C32	154.4 (11)
C6—C7—C8—C17	178.0 (4)	C30—C31—C32—C33	178.1 (13)
C7—C8—C9—C10	1.0 (6)	C31—C32—C33—C34	147.5 (16)
C17—C8—C9—C10	−177.1 (4)	C9—C10—N1—C6	0.7 (5)
C8—C9—C10—N1	−1.3 (6)	C11—C10—N1—C6	179.4 (4)
C8—C9—C10—C11	180.0 (4)	C9—C10—N1—Zn1	169.4 (3)
N1—C10—C11—C16	−48.5 (6)	C11—C10—N1—Zn1	−11.8 (5)
C9—C10—C11—C16	130.2 (5)	C7—C6—N1—C10	0.2 (5)
N1—C10—C11—C12	132.2 (4)	C5—C6—N1—C10	177.7 (3)
C9—C10—C11—C12	−49.0 (6)	C7—C6—N1—Zn1	−170.2 (3)
C16—C11—C12—C13	2.4 (8)	C5—C6—N1—Zn1	7.3 (4)
C10—C11—C12—C13	−178.4 (5)	C2—C1—N2—C5	0.1 (6)
C11—C12—C13—C14	0.2 (10)	C2—C1—N2—Zn1	172.2 (3)
C12—C13—C14—C15	−2.4 (11)	C4—C5—N2—C1	1.9 (6)
C13—C14—C15—C16	2.1 (11)	C6—C5—N2—C1	−176.5 (3)
C12—C11—C16—C15	−2.7 (8)	C4—C5—N2—Zn1	−171.2 (3)
C10—C11—C16—C15	178.0 (5)	C6—C5—N2—Zn1	10.5 (4)
C14—C15—C16—C11	0.5 (10)	C19—C20—N3—C28	177.1 (5)
C9—C8—C17—C22	21.2 (6)	C21—C20—N3—C28	−1.3 (5)
C7—C8—C17—C22	−156.7 (4)	C19—C20—N3—C29	−3.6 (9)
C9—C8—C17—C18	−161.5 (4)	C21—C20—N3—C29	178.0 (5)
C7—C8—C17—C18	20.5 (6)	C23—C28—N3—C20	1.4 (5)
C22—C17—C18—C19	0.0 (7)	C27—C28—N3—C20	−177.6 (5)
C8—C17—C18—C19	−177.3 (5)	C23—C28—N3—C29	−177.9 (5)
C17—C18—C19—C20	0.0 (8)	C27—C28—N3—C29	3.0 (9)
C18—C19—C20—N3	−179.1 (5)	C30—C29—N3—C20	−100.8 (8)
C18—C19—C20—C21	−0.8 (8)	C30—C29—N3—C28	78.4 (9)
N3—C20—C21—C22	−179.8 (4)	C1—N2—Zn1—N1	−177.6 (3)
C19—C20—C21—C22	1.6 (7)	C5—N2—Zn1—N1	−5.2 (3)
N3—C20—C21—C23	0.8 (5)	C1—N2—Zn1—I1	71.3 (3)
C19—C20—C21—C23	−177.8 (4)	C5—N2—Zn1—I1	−116.2 (3)
C20—C21—C22—C17	−1.6 (6)	C1—N2—Zn1—I2	−57.1 (3)
C23—C21—C22—C17	177.7 (4)	C5—N2—Zn1—I2	115.4 (3)
C18—C17—C22—C21	0.8 (6)	C10—N1—Zn1—N2	−170.6 (3)
C8—C17—C22—C21	178.1 (4)	C6—N1—Zn1—N2	−1.4 (2)
C22—C21—C23—C24	−1.7 (8)	C10—N1—Zn1—I1	−61.9 (3)
C20—C21—C23—C24	177.7 (5)	C6—N1—Zn1—I1	107.3 (2)
C22—C21—C23—C28	−179.3 (5)	C10—N1—Zn1—I2	89.3 (3)
C20—C21—C23—C28	0.1 (5)	C6—N1—Zn1—I2	−101.5 (2)
C28—C23—C24—C25	0.6 (6)		
